# Sex Modulates Response to Renal-Tubule-Targeted Insulin Receptor Deletion in Mice

**DOI:** 10.3390/ijms24098056

**Published:** 2023-04-29

**Authors:** Soha Sohail, Gabriella Akkawi, Taylor Rechter, Maurice B. Fluitt, Carolyn M. Ecelbarger

**Affiliations:** 1 Department of Medicine, Georgetown University, Washington, DC 20057, USA; 2 Department of Medicine, Howard University, Washington, DC 20059, USA

**Keywords:** kidney, sodium, blood pressure, metabolic syndrome, hyperglycemia

## Abstract

Insulin facilitates renal sodium reabsorption and attenuates gluconeogenesis. Sex differences in this regulation have not been well characterized. Using tetracycline-inducible Cre-lox recombination, we knocked out (KO) the insulin receptor (InsR) from the renal tubule in adult male (M) and female (F) mice (C57Bl6 background) with a paired box 8 (PAX8) promoter. Body weights were not affected by the KO, but mean kidney weights were reduced in the KO mice (13 and 3%, in M and F, respectively, relative to wild-type (WT) mice). A microscopic analysis revealed 25 and 19% reductions in the proximal tubule (PT) and cortical collecting duct cell heights, respectively, in KOMs relative to WTMs. The reductions were 5 and 11% for KOFs. Western blotting of renal cortex homogenates showed decreased protein levels for the β and γ subunits of the epithelial sodium channel (ENaC) and the sodium-potassium-2-chloride cotransporter type 2 (NKCC2) in both sexes of KO mice; however, α-ENaC was upregulated in KOMs and downregulated in KOFs. Both sexes of KO mice cleared exogenously administered glucose faster than the WT mice and had lower semi-fasted, anesthetized blood glucose levels. However, KOMs (but not KOFs) demonstrated evidence of enhanced renal gluconeogenesis, including higher levels of renal glucose-6-phosphatase, the PT’s production of glucose, post-prandial blood glucose, and plasma insulin, whereas KOFs exhibited downregulation of renal high-capacity sodium glucose cotransporter (SGLT2) and upregulation of SGLT1; these changes appeared to be absent in the KOM. Overall, these findings suggest a sex-differential reliance on intact renal tubular InsR signaling which may be translationally important in type 2 diabetes, obesity, or insulin resistance when renal insulin signaling is reduced.

## 1. Introduction

Insulin is a peptide secreted by the pancreas in response to rising blood glucose concentrations [1]. Normal insulin levels in the blood can have more than a 20-fold range in healthy (non-diabetic) humans, ranging from approximately 100 to 2000 pM depending on whether subjects are fasted or post prandial [2]. However, in insulin (receptor)-resistance, circulating insulin levels may increase at least another 2-fold [3]. A similar scenario exists in mice, with one study reporting a blood range in male mice from ~50 to 1600 pM and in female mice from 36 to 1300 pM after 4 h of fasting [4]. Therefore, the kidney, like other organs, is exposed to a wide range of insulin levels. Insulin is known to regulate the kidney in numerous ways, although many of its key roles and underlying cellular mechanisms remain obscure [5]. Moreover, little is understood regarding differences between the sexes and renal responses to insulin, as the host of earlier studies were primarily conducted in male animals, or the sex of the subjects was not reported.

Most insulin acts by binding to the classic insulin receptor (gene symbol *Insr*). Insulin resistance is thought to result primarily from a reduction in the tyrosine kinase activity of the receptor and early downstream signaling [6]. It may also result from a reduction in the protein levels of the receptor [7], impaired binding, or the overexpression of structurally related insulin-like growth factor (Igf) receptors [8,9,10]. At the whole-body level, most studies show that males (humans and animals) are more susceptible to the development of insulin resistance, despite females having a greater total fat mass [11]. In agreement with a greater InsR reserve in females, we previously showed higher renal cortical expression of the β-subunit of InsR (a subunit involved in autophosphorylation) in female mice relative to male mice (of a mixed C57Bl6/129/SV background) receiving a control diet. Nonetheless, feeding the mice with fructose increased the abundance in males but not females [12].

Insulin increases the natriferic actions of a number of sodium (Na^+^) transporters, channels, and exchangers that line the renal tubule epithelium [5,13,14,15,16]. It is important in the post-prandial reabsorption of filtered sodium in the highly regulated thick ascending limb (TAL), distal convoluted tubule (DCT), connecting tubule (CNT), and collecting duct (CD). In addition, insulin likely has a role in the handling of K^+^ by the renal tubule, although the direct regulation of distal K^+^ channels by insulin has not been fully characterized. To examine the impact of insulin in the kidney, our group and others have selectively deleted the insulin receptor (InsR) from various cell types along the renal tubule. The majority of these studies used non-inducible Cre/lox recombination that targeted the kidney, and the deletion generally occurred soon after birth, when the targeting promoters became active. We have found that deletion from the distal tubule and/or collecting duct affected blood pressure [17,18], sodium handling [17,18], and the generation of nitric oxide (NO) [17,19]. Deletion from the proximal tubule increased fasting glucose and markers of renal gluconeogenesis, at least in male mice [20,21].

Here, we use a doxycycline-inducible transgene, i.e., the paired box 8 reverse tetracycline-dependent transactivator (PAX8-rtTA) to activate deletion. In mice, PAX8-rtTA allows for the genetic targeting of the entire renal tubule in an inducible fashion [22]. It is advantageous when employed in adult mice as it removes the ability of the mice to adapt to the deletion at a young age and circumvents any developmental effects of the deletion. Our aim is to further our understanding of the role of the InsR in the kidney and to elucidate sex differences in both sodium handling/blood pressure-related responses and the gluconeogenic propensity of the renal proximal tubule.

## 2. Results

### 2.1. Protein Levels of the Insulin Receptor Were Reduced by over 70% in the KO Kidneys

**Figure 1:**
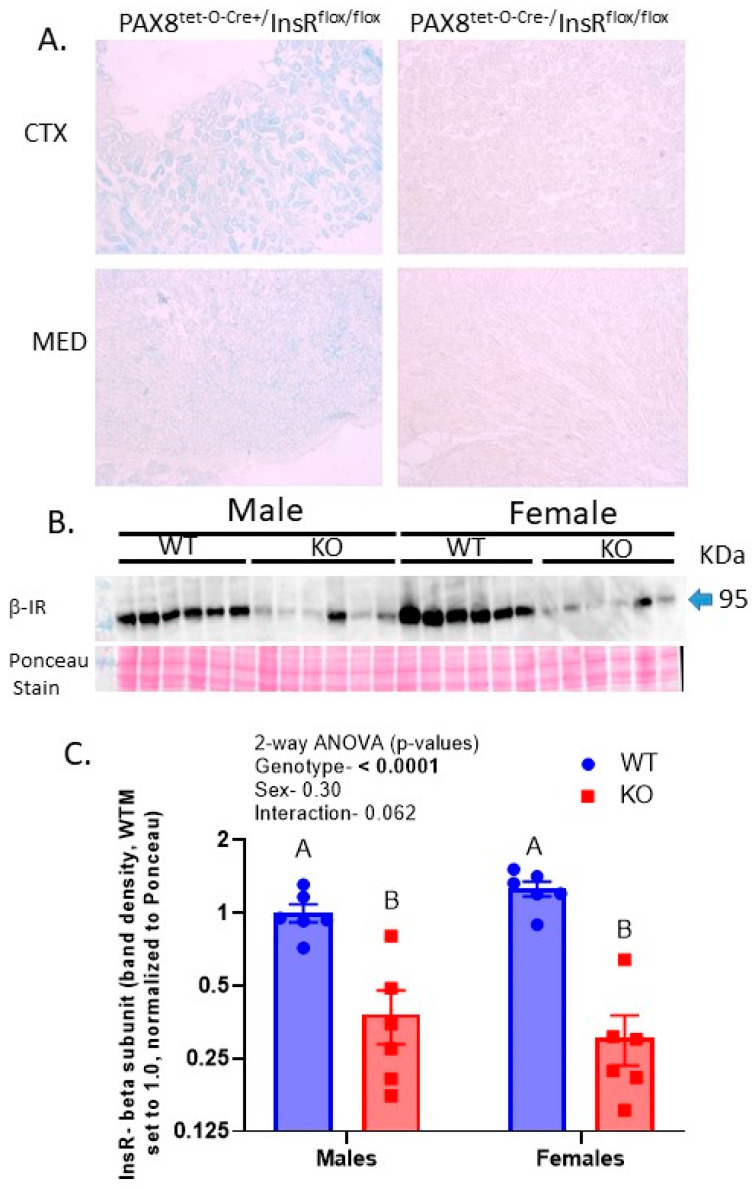
InsR knockout (KO) localization and degree in PAX8-targeted InsR KO mice—(**A**) β-galactosidase staining (indicating location of Cre-recombinase activity) in kidney cortex and medulla sections from mice that were carriers of both PAX8 and tet-O-Cre transgenes (**left panels**) or only PAX8 (**right panels**) and homozygously floxed for InsR; (**B**) Western blot of whole-kidney homogenates probed with InsR (β-subunit) antibody in WT and KO mice; (**C**) band densities of InsR Western blot (n = 6/genotype/sex, normalized to Ponceau staining); letters above bars indicate results of multiple comparison testing (MCT), with “A” significantly (*p* < 0.05) greater than “B”.

β-galactosidase activity (blue precipitate) was found in the cortical and medullary tubules, including the proximal tubules (PTs), thick ascending limbs (TAL), and collecting ducts (CD), in reporter mice that were carriers of the tetracycline-inducible (“on”) Cre-recombinase (tet-O-Cre) and PAX8 transgenes (Figure 1A). In these mice (left panels), the precipitate was absent in the vessels and glomeruli. In addition, there was no precipitate in any of the cells of the mice that were negative for tet-O-Cre but were positive for the PAX8-promoter sequence and homozygous for floxed InsR (right panels). Western blotting of the whole-kidney homogenates revealed, on average, a reduction of about 70–80% in the band density of the β-subunit of the insulin receptor (β-IR, Figure 1B,C) in both sexes of KO mice. There was no significant effect of sex on β-IR band density; however, there was a trend for the interactive term to be significant (*p* = 0.064), indicating a slightly greater level (%) of reduction in the females with KO and an approximately 25% higher mean band density in WTFs (wild-type females) versus WTMs (wild-type males).

### 2.2. Reduced Kidney Weights and Cell Height Exacerbated in KO Males

**Figure 2:**
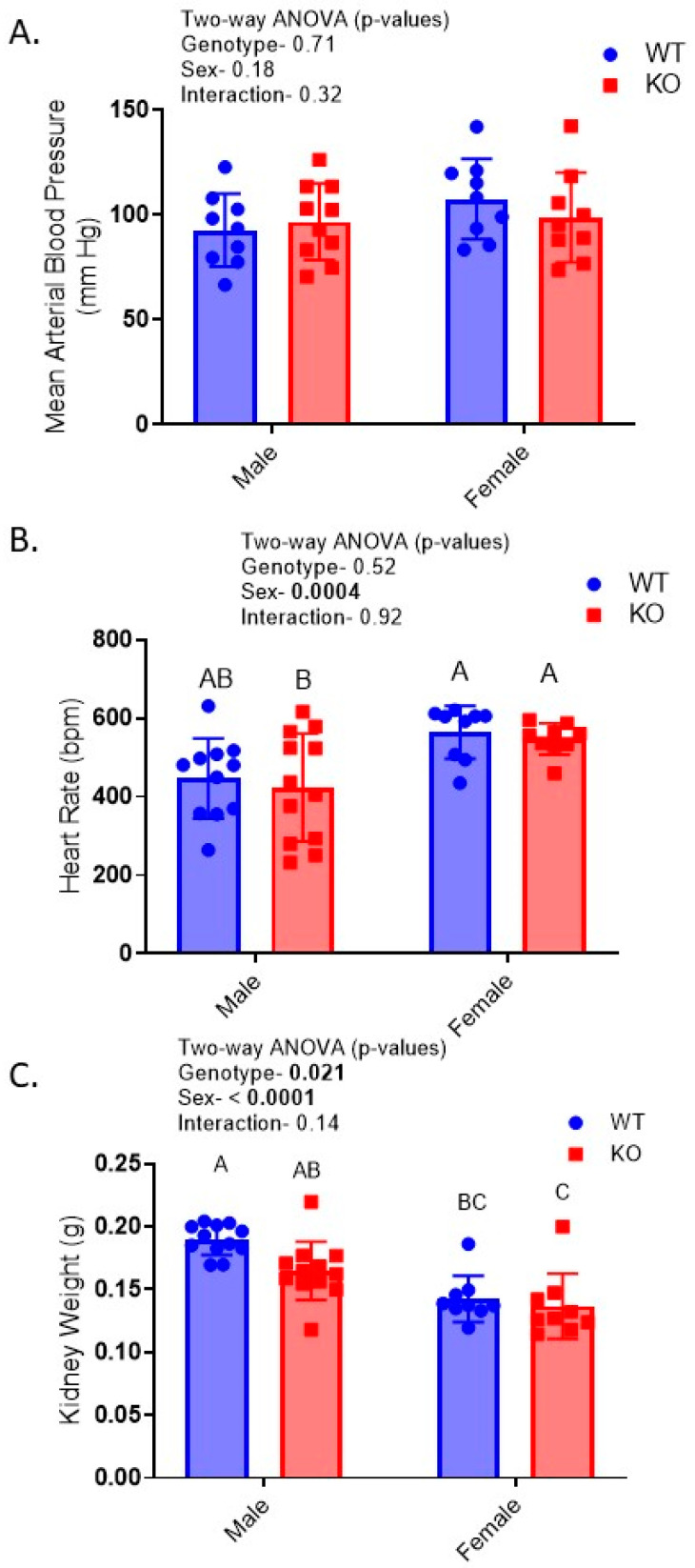
Kidney weight, blood pressure, and heart rate in PAX8-targeted InsR KO mice—(**A**) mean arterial blood pressure (MAP); and (**B**) heart rate measured by tail cuff plethysmography; (**C**) kidney wet weight (n = 9–12/genotype/sex); *p*-values from two-way ANOVA (genotype; sex) are shown within each panel; letters above bars indicate results of MCT (only conducted when a main factor *p* < 0.05), with “A” assigned to the highest mean and all means not different from it, followed by “B”, etc. Bars with letters “not in common” are significantly different from each other, e.g., “AB” versus “C”.

Conscious blood pressures were obtained in one cohort of mice just after doxycycline treatment ended. There were no significant differences due to genotype or sex for blood pressure (Figure 2A); however, heart rate was significantly higher in females (Figure 2B).

**Figure 3:**
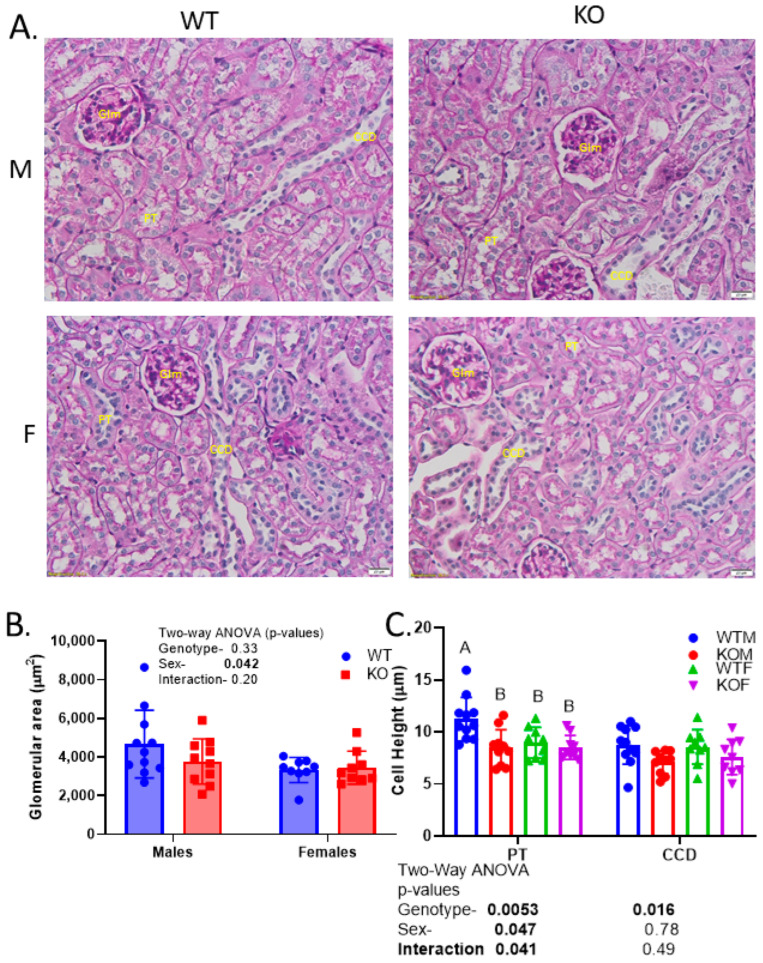
Reduced proximal tubule and collecting duct cell heights in InsR KO mice—(**A**) representative images of periodic acid–Schiff (PAS)-stained sections of kidney cortex (400×); (**B**) mean glomerular area; (**C**) mean PT and CCD cell height in WTM, KOM, WTF, and KOF mice; a two-way ANOVA (genotype; sex), followed by a multiple comparisons testing (MCT, Tukey’s), was conducted on the data (n = 9–12/genotype/sex); “A” is significantly higher than “B” by MCT; Glm—glomerulus; CCD—cortical collecting duct; PT—proximal tubule.

After the mice were euthanized, kidney wet weights were obtained and found to be significantly lower in the KO and female mice (Figure 2C). Morphometric and histologic analyses of the PAS-stained sections did not reveal any obvious differences in the degree of PAS staining (polysaccharide stain) between genotypes and sexes (Figure 3); however, KOMs (knockout males) exhibited a reduced PT cell height relative to WTMs (Figure 3A,C). The cortical collecting duct (CCD) cell height was also slightly but significantly reduced in the KO mice. The glomerular area was not different due to genotype but was lower in females (Figure 3A,B).

### 2.3. Relative Insulin-Induced Hyperkalemia in KO

In one cohort, all mice were treated with insulin (0.5 U/kg·bw) 4 h prior to being euthanized to amplify potential genotype differences in their blood chemistry (Table 1). As expected, male mice were heavier, with no effect of genotype on body weight demonstrated. Blood K^+^ levels were significantly higher in the KO mice, with the largest effect found in males. There were no genotype or sex differences in blood Na^+^, Cl^−^, bicarbonate (HCO_3_^−^), or urea nitrogen (BUN) levels.

### 2.4. Western Blotting Revealed Reduced ENaC Subunits and NKCC2 in Kidneys of the KO Mice

**Figure 4:**
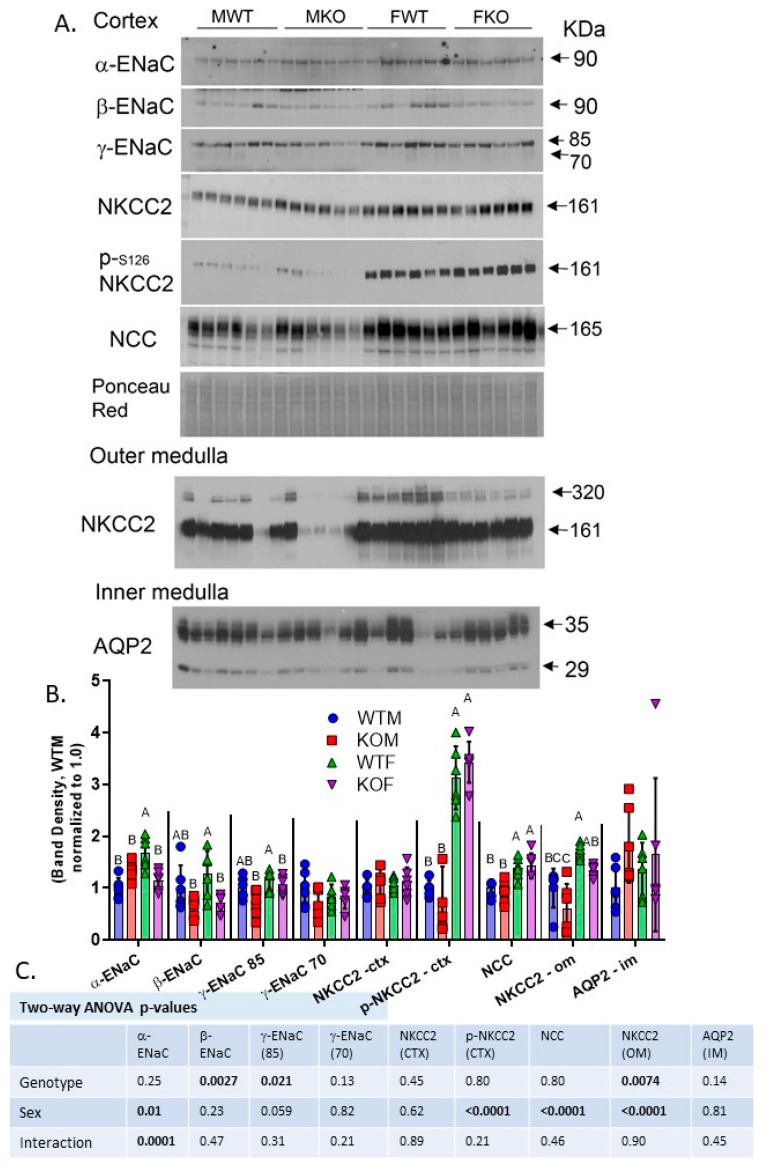
Western blotting of key sodium- and water-reabsorptive routes in kidney regional homogenates—(**A**) representative Western blots of cortex, outer medullary, or inner medullary homogenates prepared from male (M) and female (F) WT and KO mice probed with specific antibodies against the three ENaC subunits, NKCC2, NCC, and aquaporin 2 (AQP2); (**B**) summary of band densities (mean ± sem) normalized by Ponceau Red stain for all Western blots (n = 6/genotype/sex); letters above bars indicate results of Tukey’s multiple comparisons test (only conducted when a main factor < 0.05), with “A” assigned to the highest mean and all means not different from it, followed by “B”, etc. Bars with letters “not in common” are significantly different from each other, e.g., “AB” versus “C”; (**C**) summary of two-way ANOVA results (*p* < 0.05 considered significant, in bold); CTX—cortex; OM—outer medulla; IM—inner medulla.

We evaluated the renal cortex and/or medulla homogenate protein expression of three major sodium transporters and/or channels in our mice that we previously found to be regulated by insulin [16]. Representative Western blots are shown in Figure 4A. A graphic display of the band densities is shown in Figure 4B, and two-way ANOVA results are shown in Figure 4C. ENaC, which is expressed in the late DCT, CNT, and CD, was regulated by both sex and genotype. There was a strong sex × genotype interaction (two-way ANOVA) for the 90 kDa band for α-ENaC in that it was increased in the male but decreased in the female KO mice (relative to their sex-specific WTs). This band was also higher in females overall than in the males. In contrast, the 90 kDa band (major band) for β-ENaC was reduced in both male and female KO mice. Likewise, the 85 kDa band for γ-ENaC was likewise reduced in both male and female KO mice, and it was higher in females than in their male counterparts. The 70 kDa band for γ-ENaC (activating cleavage) was not affected by sex or genotype.

NKCC2, the major apical sodium, potassium, 2-chloride cotransporter of the TAL, and its activating phosphorylated form, p-serine 126 NKCC2, were evaluated in the cortex and/or inner stripe of the outer medulla. In the cortex, the band density for phosphorylated NKCC2 was more than 3-fold higher in females relative to males. There were no differences identified in cortical NKCC2 via the pan-antibody. In the outer medulla, the NKCC2 band density was significantly reduced in the KO mice and increased in the females. The major apical sodium chloride cotransporter of the DCT, NCC, was increased in female mice but was not affected by genotype. We also assessed the inner medullary abundance of the water channel, aquaporin-2 (AQP2); however, no sex or genotype differences were found for this primary apical water channel of the CD.

### 2.5. Kaliuretic Response to Diuretics Relatively Blunted in Female Mice

We next tested whether the changes we observed in the abundance of the sodium transporters/channels of the kidney were reflected in impaired or attenuated natriuretic responses to the inhibitors of these reabsorptive routes. A single i.p. injection of each transporter-select antagonist was provided to the mice in turn, with at least 3 days separating each test. Absolute excretion amounts of Na^+^ and K^+^ (4 h urine collection) and the ratio of Na-to-K in the urine are provided in Table 2. Furosemide administration (NKCC2 antagonist) resulted in a lesser kaliuretic response (absolute and relative to sodium) in females, with no genotype differences. A similar finding was observed for thiazide (NCC antagonist), although the reduction in the absolute K^+^ excreted in females did not reach significance (relative to males, *p* = 0.11). Again, no differences between genotypes were observed. No significant sex or genotype differences were observed for benzamil, an ENaC antagonist, although there was a trend for an interaction between sex and genotype (*p* = 0.055 and 0.066) for absolute K^+^ and the ratio of Na^+^-to-K^+^. That is, KOMs tended to have reduced kaliuresis, while KOsF showed slightly enhanced kaliuresis (relative to the sex-specific WTs).

### 2.6. Sex-by-Genotype Interactions in Urine Nitrates plus Nitrites (NOx), Albumin, and Glucose

**Figure 5:**
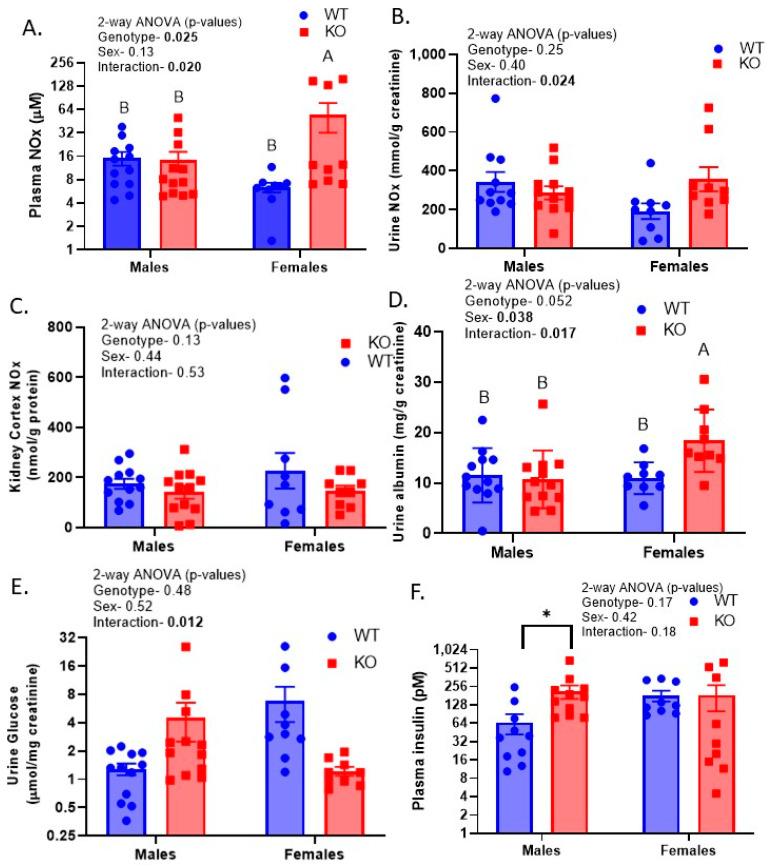
Blood and urine chemistry—(**A**) Plasma nitrates plus nitrites (NOx); (**B**) urine NOx; (**C**) kidney cortex NOx; (**D**) urine albumin; (**E**) urine glucose; and (**F**) plasma insulin (n = 8–12/group); two-way ANOVA (genotype; sex), followed by multiple comparisons test (MCT, Tukey’s), was conducted on all data sets; “A” is significantly higher than “B” by MCT; * indicates a significant difference by unpaired *t*-test.

In our previous studies, we showed that InsR deletion from the TAL via CDs, using Ksp Cadherin to drive Cre-recombinase, led to reduced nitric oxide (NO) production and excretion, which was associated with elevated blood pressure in the mice [17,19]. More recently, we demonstrated increased urine albumin in mice with the deletion of InsR from the PT [23]. We evaluated these parameters and others in these whole-renal-tubule InsR-deleted mice. Nitrates plus nitrites (NOx) were measured in the kidney cortex, plasma, and urine (Figure 5A–C). Plasma and urine NOx were both elevated to some extent in the KOFs (relative to WTFs), with either no difference (plasma) or an opposite trend (urine) observed in the males. In the case of urine, this resulted in a significant interactive term. There were no significant differences in kidney cortical NOx. Similarly, urine albumin was not significantly different between genotypes in males but was increased in the KOFs (relative to the WTFs), also resulting in a significant interactive term (Figure 5D). Likewise, there was a significant interaction for urine glucose in that it was elevated in the KOMs but reduced in the KOFs (relative to the sex-specific WTs, Figure 5E). Plasma insulin (Figure 5F) was not affected by genotype or sex when data were analyzed by two-way ANOVA; however, an unpaired *t*-test in the males revealed a significant increase (a mean increase of over 4-fold) in plasma insulin (*p* = 0.02) in the KO mice relative to the WT mice.

### 2.7. KO and Female Mice Had Lower Blood Glucose in GTT and Glutamine Challenge

**Figure 6:**
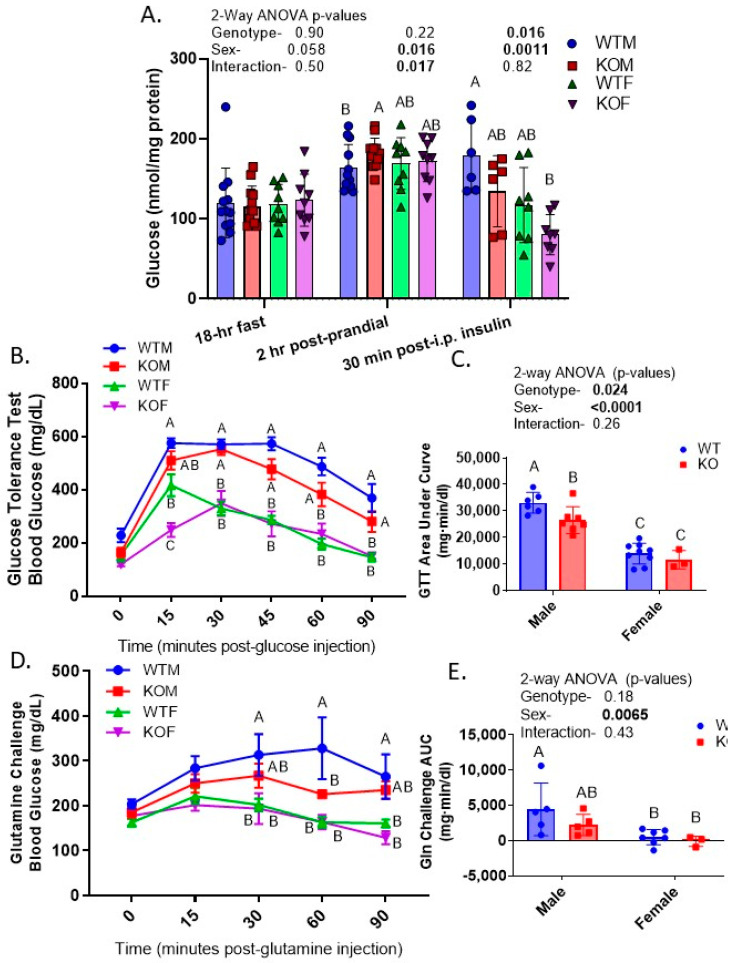
Glucose homeostasis in WT and KO mice—blood glucose (**A**) in response to 18 h of fasting; fasting followed by 2 h refeeding (re-access to chow, ad libitum); and at euthanasia 30 min after i.p. administration of insulin; (**B**) response over time to a single injection of 2 g/kg·bw glucose in the glucose tolerance test (GTT); (**C**) area under curve (AUC) for GTT; (**D**) response over time to a single injection of 2 g/kg·bw glutamine in the glutamine (Gln) challenge; (**E**) area under curve (AUC) for Gln challenge; n = 8–12/group (panel A) and 3–6/group (panels **B**–**E**); letters above bars indicate results of Tukey’s multiple comparison test (only conducted when a main factor < 0.05) with “A” assigned to the highest mean, followed by “B” then “C”. Means with letters in common, e.g., “AB” are neither different from “A” nor “B.

The metabolic state affected blood glucose differentially in the genotypes (Figure 6A). Fasting (18 h) glucose was not significantly different due to sex or genotype (Figure 5A); however, post-prandial glucose (2 h ad libitum post fast) unmasked a relative hyperglycemic spike in blood glucose in the KOMs but not the KOFs. In contrast, 30 min after an acute injection of insulin, glucose was lower in blood collected at euthanasia in KO mice of both sexes. A glucose tolerance test (GTT) was used to test how quickly mice metabolized or cleared exogenously administered glucose (Figure 6B,C). We found a significantly lower area under the curve (AUC) for glucose in the KO and female mice. The greatest difference between genotypes was observed at the 15 min time point. The glutamine challenge was provided to test the efficiency with which they converted exogenous glutamine to glucose via the process of gluconeogenesis (Figure 6D,E). Female mice of both genotypes demonstrated little increase in blood glucose when given the glutamine; however, the male mice, particularly male WT mice, had a significantly higher AUC than the females. Moreover, WTMs had significantly higher blood glucose at 60 min than the KOMs did, potentially indicating a greater ability to use glutamine as a source for gluconeogenesis.

### 2.8. Male and KO Mice Have Greater Ex Vivo Glucose-Producing Capacity of Isolated PT

**Figure 7:**
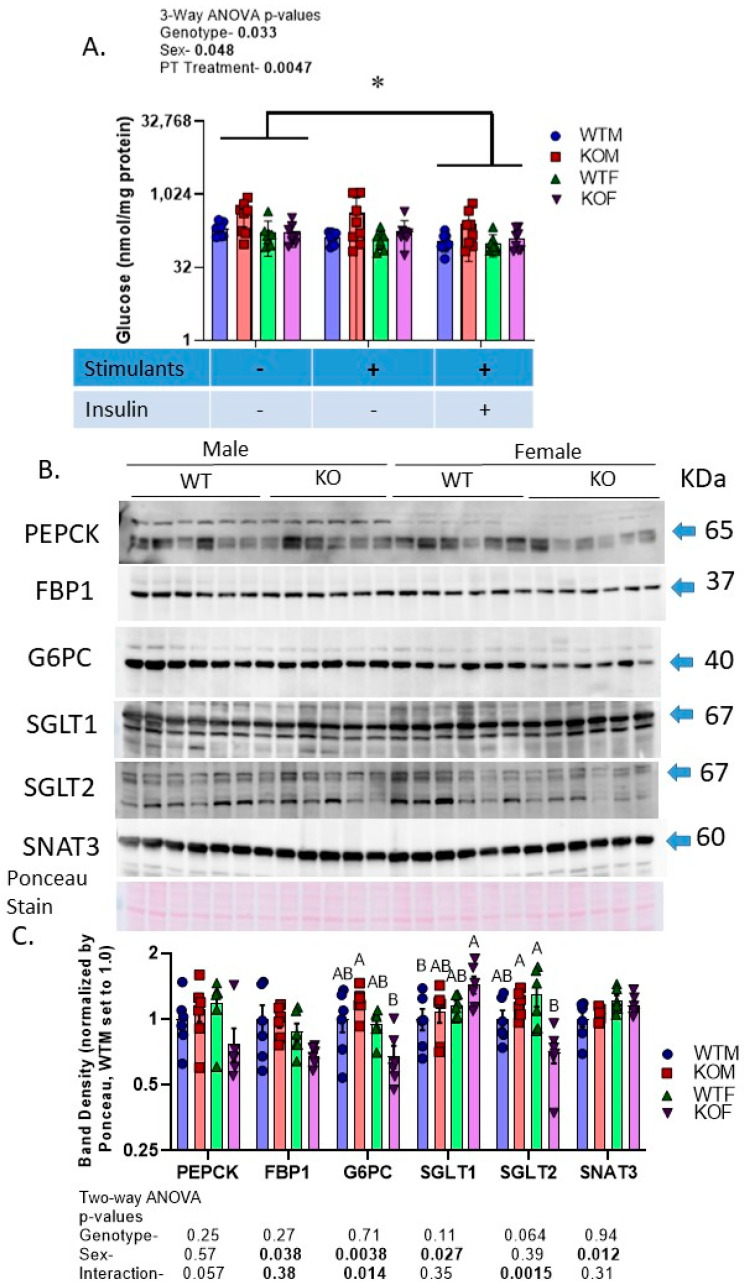
Proximal tubule gluconeogenesis and glucose transporters—(**A**) Glucose concentration in the medium from aliquots of PT-enriched suspensions (n = 6–7/mice/sex/genotype undergoing three potential ex vivo treatments in duplicate) incubated for 1 h with gluconeogenic substrates in the presence or absence of gluconeogenic stimulators, i.e., cyclic AMP and dexamethasone or insulin; * indicates a significant difference between groups (by three-way ANOVA with repeated measures for treatment); (**B**) Western blots of cortex homogenates (n = 6/group) probed with antibodies against the three rate-limiting enzymes in gluconeogenesis (PEPCK, FBP1, and G6PC), the sodium glucose cotransporters (SGLT1 and SGLT2), and the sodium-coupled neutral amino acid transporter type 3 (SNAT3); (**C**) summary (mean ± sem) of the Ponceau-Red-stain-normalized band densities for each of the blots with the mean of wild-type males (WTMs) set to 1.0; letters above bars indicate results of Tukey’s multiple comparison test (only conducted when a main factor < 0.05) with “A” assigned to the highest mean and all means not different from it, followed by “B”, etc.

In mice with InsR deleted solely from renal PTs, we found increased mRNA expression for glucose-6-phosphatase (G6P), the terminal enzyme in gluconeogenesis and modest hyperglycemia. In the current study, we extended our studies to an assessment of the ex vivo glucose-producing capacity of the PTs by measuring the glucose produced by suspended PTs over a 1 h period (Figure 7A). Suspended PTs were split into three aliquots and underwent the following treatments: (1) a vehicle in the absence of the gluconeogenic stimulants, i.e., dexamethasone and cAMP; (2) a vehicle in the presence of the stimulants; (3) 5000 pM insulin in the vehicle plus stimulants. There were significant effects of genotype, sex, and PT treatment on the glucose measured in the supernatant. KO (primarily male) and male mice, in general, demonstrated a significantly greater production of glucose (per mg of protein in the aliquot). The addition of insulin suppressed the production of glucose. There was no effect of the stimulants. To determine whether the genotype and sex differences in the blood and urine glucose levels and in the glucose-producing capacity of the PT corresponded with changes in the protein levels in the kidney, we used Western blotting (Figure 7B,C). The levels of sodium glucose cotransporter type 1 (SGLT1) were increased in female mice (especially KOFs). SGLT2, on the other hand, was reduced in KOFs, with a significant interactive term (*p* = 0.015). There was no effect of sex or genotype on the protein levels of PEPCK or FBP1; however, G6PC (a catalytic subunit of G6P), was oppositely regulated in male and female KO mice, showing upregulation in the KOMs and a reduction in the KOFs. Female mice had a slightly greater abundance of the sodium-coupled neutral amino acid transporter type 3 (SNAT3), the primary basolateral glutamine transporter, than the male mice did, with no differences due to genotype. 

### 2.9. Testing of Leakiness in Transgene Expression

Tetracycline-inducible Cre-recombinase constructs have been reported to have a degree of leakiness [24]. We tested this in our systems using two approaches. In the first approach, we evaluated select indices of sodium and glucose handling/metabolism in genetically WT (negative for PAX8, tet-Cre, or both) and KO (positive for both transgenes) littermates prior to doxycycline treatment. These data are shown in Table 3. We detected one genotype difference in that the natriuretic response to furosemide was enhanced in the genetic KO mice. This may indicate upregulated NKCC2, although additional studies would be warranted. In the second approach, in an additional set of male mice, we harvested kidneys from genetic KO and WT mice that had never been exposed to doxycycline (Supplemental Figure S1). The genetic KO mice did not show any reduction in the band density for the β-subunit of InsR in cortex or medulla homogenates on Western blots when compared to their WT littermates. Taken together, although we saw minimal differences prior to doxycycline, additional studies are warranted to examine the leakiness of the system more closely.

## 3. Discussion

Insulin has been shown to have pleiotropic actions along the renal tubule that generally involve regulating the reabsorption, metabolism, or production of nutrients [5]. Little has been done to ferret out sex differences in these responses, although there are numerous known differences in kidney function between the sexes [25]. The current set of studies provide novel insights into the importance of InsR signaling along the renal tubule, particularly sex differences in the role of this receptor.

Sex differences in renal responses to insulin may be an important determinant of how the kidney adjusts to states of hyperinsulinemia, e.g., during metabolic syndrome (MetS). Due to their novelty, we will principally discuss the significant interactions between genotype and sex that we observed in our model system via two-way ANOVAs. In general, the renal-tubule-targeted InsR KO mouse has a mild phenotype when in the basal state; this is in agreement with the elegant studies by Nizar et al., which used a similar mouse model [26]. Our current studies also build upon former work from our own laboratory, which examined the impact of non-inducible KO of the InsR from specific cells along the renal tubule, including from the collecting duct (CD) principal cells (using aquaporin 2 (AQP2)-promoter) [18], the thick ascending limb through the CD (using kidney-specific cadherin (Ksp)-promoter) [17,19], and the proximal tubule (using γ-glutamyltransferase, (gGT)), to target Cre-recombinase activity [20,23]

Starting with changes in kidney weight and cell height, although both male and female KO mice demonstrated reduced kidney weight relatives to same-sex WT mice, the reduction in KO mice was greater in males. The wet weight of the kidneys was reduced, on average, by 13% in males versus 3% in females. Similarly, the PT and CCD mean cell heights were reduced to a greater extent in the KOMs relative to the KOFs. We did not measure the cell height of the TAL as it was difficult to determine morphologically with our approach. These reductions in kidney wet weight are in line with what we reported previously in mice with γGT-targeted InsR/insulin-like growth factor type 1 receptor (Igf1R) dual KO [27]. In that study, we also determined that the KO increased the DNA-to-protein ratio (smaller-sized cells). In general, the current study confirmed our previous work [12], showing a slightly higher (20–30%) expression of InsR protein in female mice relative to male WT mice; however, these values reflect the normalization of the band density to the protein load. The female kidneys were also lower in weight overall (and when compared to body weight) when compared to male kidneys; thus, the relatively reduced expression in males may be, at least in part, a dilutional effect of the other, more abundant proteins.

Changes in the size of the cells, especially in the PT, can affect reabsorptive capacity. A morphometric analysis of the kidney using low-power micrographs by Harris and associates [28] revealed that the PT volume encompassed 60 versus 42% of the cortex in male and female C57Bl6 mice, respectively. Thus, assuming a similar expression of transporters/channels, the reabsorptive capacity of the PT in male mice may be higher. Despite this predicted increase in the PT’s reabsorptive capacity in males, we found that urine glucose (mg/g creatinine), which is predominantly reabsorbed in the PT, was generally increased in knockout males (KOMs) but decreased in knockout females (KOFs) relative to their same-sex wild-types (WTs). The increase in males agrees with the findings of Nizar et al. (also in PAX8-targeted renal InsR KO mice) with respect to glucosuria, at least under some conditions, in the KOMs [26]. They also showed a reduction in mRNA and, to some extent, the protein expression of sodium-glucose cotransporter type 2 (SGLT2/*Slc5a2*) in the kidneys of their KO mice but only when the mice were co-administered fludrocortisone (a mineralocorticoid agonist). In agreement, we demonstrated a reduction in SGLT2; however, in our case (studying both sexes under basal conditions), we found the decrease solely in the KOFs. In addition, we found an increase in SGLT1 in the KOFs, which may represent a compensatory upregulation to reduce glucosuria since SGLT1 is expressed in more distal parts of the PT (as compared to SGLT2) [29]. Whether the upregulation of SGLT1 in the KOFs but not in the KOMs accounted for the sex difference in urine glucose will require additional study.

Another factor which may have enhanced urine glucose excretion in the KOMs but not the KOFs was the relatively greater effect of the KO on the glucose-producing capacity of the PT in the males. We found that KOMs but not KOFs demonstrated increased glucose production ex situ in isolated PTs (compared to their respective WTs). This agrees with our previously reported studies using mice with an InsR KO in a non-inducible fashion from the PT using γ-glutamyltransferase (gGT) to target the deletion [20]. Those mice showed increased activity and mRNA expression of G6PC in the kidney, as well as modestly elevated fasting blood glucose levels. Unfortunately, the studies were conducted only in male mice. Additional studies conducted in primary human PT cells showed an upregulation of PEPCK and G6PC mRNA as well as glucose production after siRNA knockdown of the InsR [21]. Upregulation of one or more of the rate-limiting enzymes in gluconeogenesis is likely due to the reduced phosphorylation of the forkhead box protein O1 (FOXO1) [30,31], which has been demonstrated to play a role in enhanced hepatic gluconeogenesis in the liver via the reduced phosphorylation of FOXO1. This allows the transcription factor to translocate to the nucleus and increase the expression of gluconeogenic enzymes.

The current experiments did not show any sex or genetic differences in the renal abundance of PEPCK or FBP1; however, G6PC was reduced in the KOFs (relative to WTFs). This decrease was absent in the KOMs; in fact, they showed a modest increase (consistent with our PT-select KO mice) [20]. We have also reported lower G6PC renal protein levels in mice with a PT-targeted dual InsR/Igf1R KO [32]. In that case, the reduction occurred in both the male and female KO mice. Thus, there may be two countering influences of renal InsR deletion on the expression of G6PC, with the deletion enhancing G6PC via greater FOXO1 transcriptional activity that is more prominent in the males and a generalized reduction (possibly due to the reduced PT size) in both sexes.

Taken together, it appears that the insulin regulation of renal glucose homeostasis is quite different between the sexes. A sharp reduction in InsR signaling in the kidney of male animals appears to be more likely to lead to hyperglycemia (due to enhanced gluconeogenesis and a smaller decrease in G6PC and SGLT2). These adaptive changes do not happen in females; thus, they upregulate SGLT1 to preserve filtered glucose but are not as subject to hyperglycemia. Whether this is translationally important to humans is not known, but it is something to consider when evaluating T2D persons who may have reduced renal insulin sensitivity [33,34,35].

Another area in which InsR signaling along the renal tubule has proven to play a modulatory role is in the regulation of renal sodium reabsorption [5]. Moreover, there are a host of sex differences in the regulation of renal sodium transporters and channel subunits; perhaps the most encompassing differences are a tendency for greater Na^+^ reabsorptive potential in the PT in males and a greater relative activity in the TAL, DCT, and CD in females [36,37,38]. In this model, consistent with our AQP2-targeted InsR KO mice [18] and our AQP2-targeted mechanistic target of rapamycin (mTOR) KO mice [39], we observed reduced protein levels of one or more of the ENaC subunits in the KO mice. Regarding the abundance of α-ENaC, a subunit of the channel that is highly upregulated by aldosterone [40], there was a significant sex-by-genotype interaction in that the abundance was reduced in the KOFs but was marginally increased in the KOMs relative to their respective WTs. In agreement with this, there was a trend for a significant interaction between sex and genotype for benzamil sensitivity (an indirect measure of in vivo ENaC activity) in that natriuresis was enhanced in KOMs (indicative of higher ENaC activity), but not changed in KOFs. The β- and γ-subunits, in contrast, were reduced by about 15% on average in each sex in the KO mice.

The sodium (potassium)-coupled cotransporters (NKCC2 and NCC) were also differentially regulated between the sexes, with higher levels of both in female mice. In fact, we found a remarkable 3–4-fold higher abundance of the phosphorylated (serine 126, activating) form of NKCC2 (thick ascending limb transporter) in female mice of both genotypes. A recent study showed this phosphorylated form of NKCC2 was increased in obese mice [13]. Veiras et al. [41] demonstrated a 20% (significant) increase in another phosphorylated form of NKCC2 (threonine 96/101) in female rats, supporting the more distal shift in sodium reabsorption in females relative to males in general (at least in rodents). NKCC2 was also significantly reduced in the outer medullary homogenates (medullary thick ascending limb, TAL) in the KO mice. Whether a reduction in the size of the TAL cells in the KO mice was a determining factor or simply a reduction in the number of transporters per cell cannot be determined at this point. The diuretic tests also revealed an interesting sex difference that was independent of genotype: female mice had higher urine Na-to-K ratios in both thiazide and furosemide tests. In other words, these medications were more “K^+^ sparing” in the female sex. It may be that males activated ENaC to a greater degree in response to furosemide and thiazide in an attempt to reabsorb the higher Na^+^ load. An increase in ENaC activity would reduce the Na^+^-to-K^+^ ratio in urine through its exchange at this site. However, this is unclear at this point. Nonetheless, male but not female KO mice showed relative hyperkalemia (compared to sex-specific WTs), suggesting the males were more sensitive to the disruption of K^+^ homeostasis via renal InsR deletion. Insulin may regulate K^+^ secretion at a number of points along the renal tubule, and it is also essential in cellular internalization of K^+^ within the body [41].

Finally, we found strong sex-by-genotype interactions for nitric oxide (NO) regulation and urine albumin. Previously, we showed reduced urine NOx (nitrates plus nitrites) coupled to a slightly higher blood pressure in the Ksp-targeted InsR KO mice [17]. In the current study, urine and plasma NOx were marginally but not significantly reduced in the KOMs, but the KOFs actually showed a significant increase in these measures, with an increase in urine NOx of about 70% and a plasma NOx value over 7-fold higher. The source of the increased urine and plasma NOx in the KOFs was not likely the kidney, as there was a tendency for renal (at least cortical) NOx to be suppressed in both male and female KO mice. In addition, urine albumin was higher in KOFs relative to the WTFs. We previously showed elevated urine albumin in the γGT-targeted InsR KO male mice [23]. We suspect this may be due to impaired albumin reuptake in the PT in the KO mice, as we found some reduction in megalin and cubulin abundances and apical labeling in our previous study.

## 4. Materials and Methods

### 4.1. Generation of the Knockout

Mice with an insulin receptor knockout (InsR KO) targeted specifically to the renal tubule cells were generated by crossing mice with loxP sites flanking the InsR gene [42] with mice possessing tetracycline-inducible Cre-recombinase driven by the paired box 8 (PAX8)-promoter [22], which were obtained from the colony of David Ellison (Oregon Health & Science University). After two crosses, ensuring that all mice were homozygously floxed for InsR, future generations of mice were genotyped for the presence of both tetracycline-inducible Cre-recombinase and the PAX8 promoter transgenes, which segregate independently. The mice that contained both genes were “KO”, and those that were negative for PAX8, Cre, or both transgenes were used as wild-type (WT) mice. To activate Cre-recombinase and induce KO, all mice were fed a doxycycline-containing diet (Purina Rodent Chow 5001 with 200 ppm Doxycycline, Research Diets) for a minimum of two weeks. Mice were maintained on the C57Bl6 background strain. For the experiments, 3–5 month old male and female mice were used. The mice were humanely euthanized under thiobutabarbital (Inactin, Sigma Aldrich, St. Louis, MO, USA) or isoflurane (Patterson Veterinary Supply Inc., Bessemer, AL, USA) anesthesia. Heparinized blood was drawn from the heart, and the kidneys were rapidly collected and weighed prior to sample preparation. In some cases, the kidneys were perfused with room temperature phosphate-buffered saline (PBS) prior to harvest and then fixed with 4% paraformaldehyde and embedded in paraffin. Paraffin sections (5 μm) were prepared by the Georgetown University Histopathology & Tissue Shared Resource.

To determine the cellular and tubular locations of the activity of Cre-recombinase, we also crossed KO female mice with male mice that were homozygous for a transgene of the LacZ gene, including a DNA stop sequence flanked by loxP sites (The Jackson Laboratory, Bar Harbor, ME, catalog #002073 B6;129-Gtrosa26tm1Sor, Soriano Line). Cre-recombinase actively removes the stop sequence, allowing for the expression of β-galactosidase, an enzyme that produces a blue precipitate when a substrate is provided. The activity of β-galactosidase was evaluated in kidney slices ex vivo after harvesting the kidneys from euthanized mice after 2 weeks of doxycycline treatment.

### 4.2. Kidney Sample Preparation and Western Blotting

The right and left kidneys were dissected into the cortex and medulla, and each part was minced and prepared in Laemmli sample buffer for Western blotting, as previously described [43]. In some cases, the left kidney was coronally bisected, and half was placed into 4% paraformaldehyde for fixation prior to paraffin embedding for histology. Primary antibodies used for Western blotting were as follows: (1) InsR-β (A303–712A, polyclonal rabbit, Bethyl Laboratories, Montgomery, TX, USA); (2–4) α-, β-, and γ-ENaC (our own rabbit polyclonals) [18]; (5–7) NKCC2, AQP2, and NCC (our own rabbit polyclonals) [12]; (8) p-126-serine NKCC2 (rabbit polyclonal, a kind gift from Mark Knepper); (9) PEPCK (sc271019, rabbit polyclonal, Santa Cruz Biotechnology, Dallas, TX, USA); (10) FBP1 (109020, monoclonal rabbit, Abcam, Waltham, MA, USA); (11) G6PC (PAS-42541, polyclonal rabbit, Invitrogen, Waltham, MA, USA); (12) SGLT1 (NBP2-20338, polyclonal rabbit, NovusBio, Littleton, CO, USA); (13) SGLT2 (ab37296, polyclonal rabbit, Abcam), and (14) SNAT3 (14315, polyclonal rabbit, Proteintech, San Diego, CA, USA).

### 4.3. Kidney Morphometry

Kidney sections were stained with periodic acid–Schiff stain and evaluated for morphometric histologic differences using an Olympus Microscope (BX43). The mean glomerular areas and cell heights of cortical collecting ducts (CCDs) and proximal tubules (PTs) were determined at 400× magnification, using cellSens software, V4.1 (Olympus, Shinjuku, Tokyo, Japan). The analyzer was blinded to the genotype and sex of the stained section while obtaining the measurements. Three measurements were made for the representative glomerular, PT, and CCD and were averaged for that mouse.

### 4.4. Blood and Urine Chemistry

Urine (24 h) was collected from mice while singly housed in mouse metabolic cages (Hatteras Instruments, Cary, NC, USA). The mice had ad libitum access to food and water during urine collection. Urine was collected in tubes containing 50 μL of pre-added mineral oil and 50 μL of an antibiotic cocktail (Antibiotic/Antimycotic, Gibco, Billings, MT, USA) to reduce bacterial growth. Blood chemistry was measured using fresh blood collected during euthanasia via an iSTAT Handheld Blood Analyzer (Abbott, Chicago, IL, USA) and EC8+ cartridges. Creatinine in the urine and nitrites plus nitrates (NOx) in the urine, kidney cortex, and plasma were analyzed via colorimetric assays (Cayman Chemical Company, Ann Arbor, MI, USA). Albumin in the urine was analyzed via an ELISA (Crystal Chem, Elk Grove Village, IL, USA).

### 4.5. Blood Pressure and Insulin Administration

In some sets of mice, blood pressure and heart rate were measured non-invasively via tail cuff plethysmography (CODA^®^ High Throughput System, Kent Scientific Corporation, Torrington, CT, USA). Non-anesthetized mice were gently secured by hand in a plastic restraining tube with a small hole for the nose at the front. They were then covered with a black blanket and warmed to about 35 °C on a heated platform. The tail cuff and volume pressure-recording sensor were placed midway up the tail. After warming the mice and allowing them to relax for about 5 min, measurements were taken (20 consecutive). The procedure lasted about 15 min. The mice were then placed back into their home cages. In some studies, as noted, mice were injected intraperitoneally (i.p.) with insulin (Humulin-R, Eli Lilly, Indianapolis, IN, USA) in sterile saline prior to euthanasia to enhance genotypic differences.

### 4.6. Natriuretic Tests

The natriuretic response to a single i.p. injection (0.2 mL/25 g mouse) of benzamil (1.4 mg/kg·bw), furosemide (12 mg/kg·bw), or thiazide (7.5 mg/kg·bw) diluted in water was used to gauge the in vivo activity of ENaC, NKCC2, and NCC, respectively, in the mice. After the injection, urine was collected in mouse metabolic cages (Hatteras Instruments) for 4 h. After recording the volume, the urine Na^+^ and K^+^ concentrations were measured with either an EasyLyte Electrolyte Analyzer (Medica Corporation, Bedford, MA, USA) or a flame photometer (BWB-XP flame photometer, BWB Technologies, Newbury, UK).

### 4.7. Glucose Tolerance and Glutamine Challenge

A glucose tolerance test (GTT) was conducted by obtaining a baseline blood glucose reading from tail blood (1 mm snip off the end of the tail) with a glucometer (Oh’ Care Lite, Blood Sugar Monitor and Strips). Next, the mice were i.p. administered 2 g/kg·bw glucose in a 20% solution in water. The blood glucose was measured 15, 30, 45, and 90 min post injection by disturbing the light scab on the tail wound. In a separate set of mice, a glutamine challenge was conducted in which glutamine (Gibco™ 200 mM solution) was provided i.p. at 2 g/kg·bw. Glutamine is a primary substrate for renal but not liver gluconeogenesis [44]. In this test, the blood glucose was measured at 15, 30, and 90 min post injection. Mice were fasted for 6 h prior to each test.

### 4.8. Proximal Tubule (PT) Suspensions and Gluconeogenesis

A proximal-tubule-enriched suspension was prepared from the kidney cortex, as previously described [32]. Briefly, minced cortices were digested with collagenase for approximately 30 min while heating to 37 °C and periodically mechanically disrupting the tissue by pulling it up and down into a Pasteur pipet. A PT-enriched fraction of the suspended tubules was obtained via Percoll gradient centrifugation [45]. The tubules were aliquoted in duplicate and treated with insulin (5000 pM) or a vehicle (Dulbecco’s modified eagle’s medium (DMEM, Gibco™) without glucose), plus or minus dexamethasone and cyclic AMP (gluconeogenic stimulators).

### 4.9. Statistics

The data were analyzed primarily via two-way ANOVA (sex and genotype were the main factors) using Graphpad Prism (Version 9) software, followed by multiple comparison testing amongst all four groups (Tukey’s test). *p*-values (or adjusted *p*-values) less than 0.05 were considered significant. A three-way ANOVA (sex; genotype; treatment) with repeated measures for treatment was used to analyze glucose in the medium from PT suspensions with paired aliquots from the same mice undergoing three potential treatments. An unpaired *t*-test was also used for plasma insulin between the male groups as females had higher variability.

## 5. Conclusions

Overall, female and male renal-targeted KO mice responded and/or adapted to the deletion of the InsR in different fashions. While both sexes showed sensitivity to the loss of the InsR, the phenotype in males suggested a greater enhancement of PT gluconeogenesis, with subsequently higher plasma insulin. In contrast, KOFs demonstrated more generalized effects, with greater deficiencies in the PT transport(ers) albumin and SGLT2 and elevations in systemic and urine nitric oxide. Thus, it is possible that renal deficiencies in InsR signaling may be expected to be a greater driver of progression to type 2 diabetes in males when compared to females, while females may present earlier with electrolyte/blood chemistry disorders.

## Figures and Tables

**Table 1:** Body weight and blood chemistry *.

Group	Body Weight (g)	Hct (% RBV)	Blood Na^+^ (mM)	Blood K^+^ (mM)	Blood Cl^−^ (mM)	Blood HCO_3_^−^ (mM)	BUN (mg/dL)
WTM	33.5 ± 1.1 ^A^	29.5 ± 1.4	145 ± 3	3.5 ± 0.4 ^B^	117 ± 2	22.8 ± 0.7	19.2 ± 4
KOM	34.2 ± 1.2 ^A^	29.7 ± 1.4	141 ± 1	5.2 ± 0.5 ^A^	118 ± 2	24.8 ± 1.2	24.8 ± 0.3
WTF	24.4 ± 1.1 ^B^	29.1 ± 1.6	144 ± 2	4.6 ± 0.3 ^AB^	120 ± 2	21.6 ± 1.7	15.6 ± 1.4
KOF	24.4 ± 2.0 ^B^	29.5 ± 0.9	144 ± 2	4.7 ± 0.3 ^AB^	121 ± 1	22.5 ± 0.5	19.2 ± 1.3
2-way Analysis of Variance (Genotype × Sex)							
G	0.84	0.88	0.44	**0.035**	0.52	0.43	0.17
Sex	**<0.0001**	0.88	0.76	0.53	0.28	0.35	0.17
I	0.80	0.95	0.41	0.051	0.99	0.78	0.76

* mean ± sem (n = 6–8/group); non-fasted; 4 h after 0.5 U/kg·bw insulin, i.p.; Superscript letters assigned based on Tukey’s multiple comparisons test—“A” greater than “B” but not “AB” etc.; *p* < 0.05 considered significantly different; WTM—wild-type male; KOM—knockout male; WTF—wild-type female; KOF—knockout female; RBV—red blood cell volume; G—genotype; I—interaction.

**Table 2:** Responses to acute natriuretics *.

	Furosemide			Thiazide			Benzamil		
Group	Na^+^ †	K^+^ †	Ratio (Na/K)	Na^+^ †	K^+^ †	Ratio (Na/K)	Na^+^ †	K^+^ †	Ratio (Na/K)
WTM	115 ± 23	103 ± 31	1.37 ± 0.12	59 ± 19	98 ± 39	0.74 ± 0.08	68 ± 11	36 ± 14	2.8 ± 0.4
KOM	119 ± 18	72 ± 10	1.88 ± 0.29	85 ± 19	112 ± 37	0.86 ± 0.07	48 ± 9	15 ± 7	5.6 ± 1.3
WTF	105 ± 12	53 ± 8	2.16 ± 0.20	51 ± 8	50 ± 9	1.24 ± 0.16	69 ± 9	19 ± 2	3.7 ± 0.6
KOF	95 ± 15	52 ± 13	2.02 ± 0.26	69 ± 17	67 ± 21	1.17 ± 0.23	69 ± 12	22 ± 4	3.4 ± 0.6
2-way Analysis of Variance (Genotype × Sex)									
G	0.69	0.28	0.11	0.11	0.30	0.84	0.57	0.40	0.12
Sex	0.26	**0.034**	**0.0073**	0.78	0.17	**0.019**	0.19	0.67	0.46
I	0.55	0.50	0.64	0.36	0.51	0.65	0.23	0.066	0.055

* mean ± sem (n = WTM—8–10; KOM—9–12; WTF—11–12; KOF—9–12); † μmol/30 g·bw/4 h; *p* < 0.05 considered significantly different; Tukey’s multiple comparisons test revealed no significant differences between individual pairs of means (with padj for multiple comparisons); G—genotype; I—interaction.

**Table 3:** Key measures pre-doxycline in genetically WT and KO mice *.

Group	Body Weight (g)	MAP (mm Hg)	GTT (g·min/dL)	Fasting Glucose (mg/dL)	Furosemide Test Na^+^ †	Thiazide Test Na^+^ †	Benzamil Test Na^+^ †
WTM	30.2 ± 1.6 ^A^	104 ± 8	24.5 ± 3 ^A^	108 ± 16 ^A^	55± 14	23 ± 7 ^B^	50 ± 15
KOM	28.7 ± 1.5 ^A^	104 ± 5	25.1 ± 1 ^A^	102 ± 8 ^A^	87± 15	17 ± 6 ^B^	46 ± 10
WTF	22.6 ± 1.6 ^B^	102 ± 13	18.4 ± 1 ^A^	83 ± 5 ^B^	71 ± 12	45 ± 8 ^A^	67 ± 13
KOF	24.7 ± 2.0 ^B^	104 ± 6	17.5 ± 2 ^A^	81 ± 8 ^B^	101 ± 12	68 ± 16 ^A^	71 ± 19
2-way Analysis of Variance (Genotype × Sex)							
G	0.76	0.11	0.93	0.51	**0.030**	0.40	0.82
Sex	**<0.0001**	0.19	**0.0013**	**0.0043**	0.27	**0.0011**	0.097
I	0.37	0.23	0.70	0.67	0.93	0.17	0.83

* mean ± sem (n = 6–9/sex); † μmol/30 g·bw/4 h; means with superscript “A” are significantly higher (padj < 0.05) than “B” by Tukey’s multiple comparison test following two-way ANOVA; G—Genotype.

## Data Availability

Raw data sets are available upon request from the corresponding author.

## Supplementary Materials

The following supporting information can be downloaded at: https://www.mdpi.com/article/10.3390/ijms24098056/s1.
