# Supplementary material for: Sex Modulates Response to Renal-Tubule-Targeted Insulin Receptor Deletion in Mice
Source: Int J Mol Sci. 2023 Apr 29;24(9):8056. doi: 10.3390/ijms24098056 (PMC10178497; doi:10.3390/ijms24098056)
Supplement: Supplementary file 1 [file ijms-24-08056-s001.zip › ijms-2283445-supplementary.pdf]

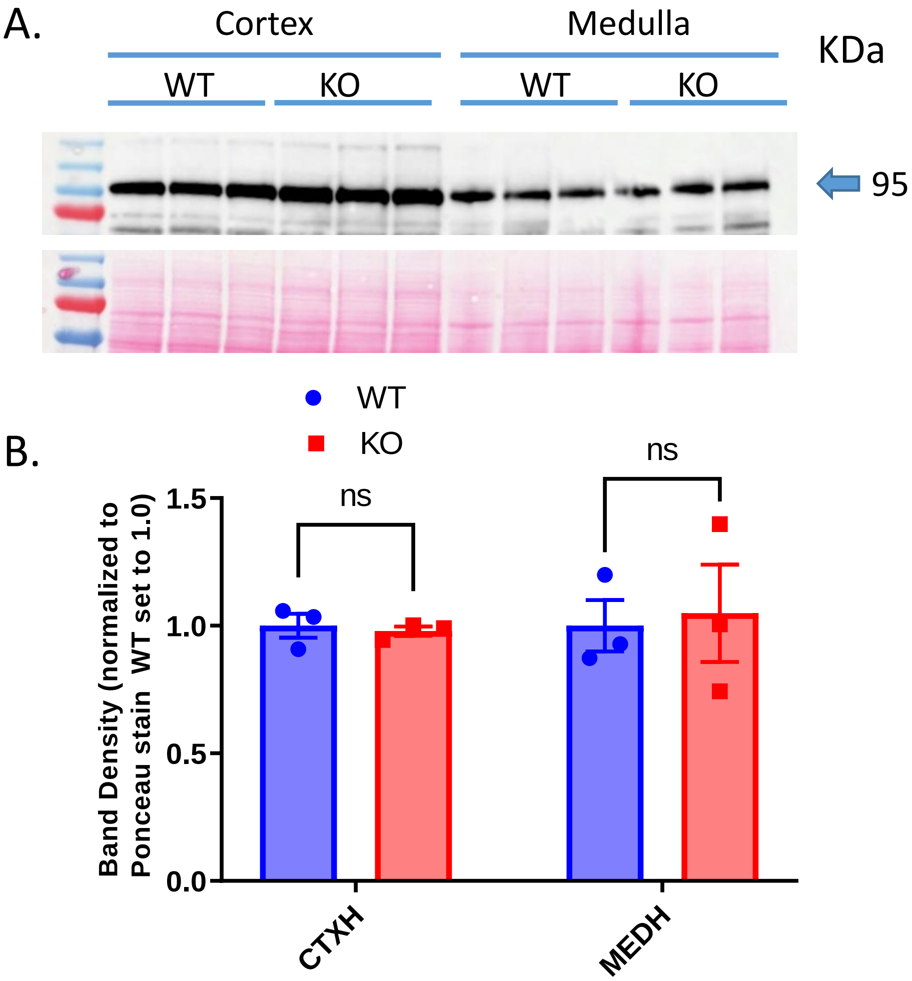

Figure S1. No reduction in InsR-β subunit protein in genetically-KO mice in the absence of doxycycline treatment- A. western blot of kidney cortex (CTXH) and medullary (MEDH) homogenates from male genetic WT (floxxed InsR/negative for tet-O-Cre and/or PAX8) or KO (floxxed InsR/positive for both tet-O-Cre and PAX8 transgenes); unpaired t-test within each region; 40 μg protein loaded/lane/sample.
